# The Involuntary Action of the Nervous System

**Published:** 1877-09

**Authors:** John J. Caldwell

**Affiliations:** Baltimore, Md.


### THE INVOLUNTARY ACTION OF THE NERVOUS SYSTEM.

BY JOHN J. CALDWELL, M. D., BALTIMORE, MD.

*Read before the American Dental Convention, Oakland, Md. August 17th, 1877.*

"The Brain not the Sole organ of the mind, for wherever gray nerve matter functionates, mind is a resultant.—HAMMOND.

"We can no more stop thinking than we can stop breathing."
PENDLETON.

"There is nothing great but man; there is nothing great in man but mind."—HAMILTON.

"Soul, immortal as its sire, can never die."

In the words of the great Physiologists, Carpenter, Draper, and Dalton, the great sympathetic distributes its nerve force, to organs, over which, consciousness and the will have no immediate control, for instance the functions of the heart, kidney, and liver.

The focusing of the light in the eye; the dilation and contraction of the pupil; the protection of the olfactory membrane, relaxing and stretching the tympanum; dilating and contracting the vascular system and the government of nutrition and temperature, etc.

As a typical illustration of involuntary force we will cite its influence on the heart's action—say for seventy years—and, for convenience fix the pulsations at 60 per minute. Then we will have the proposition of 60 x 60=3,600 beats per hour. Again 3,600 multiplied by ten equals 36,000 beats in ten hours; or in the words of the learned Draper, this little organ can execute "three thousand million beats without a stop; and propel a half million tons of blood, and momentarily wasting, repairs its own waste all this time."

The mathematical rhythm of four moving cavities, the perfect closure of its mitral and semi-lunar valves, and the regurgitating play of its tricuspid, have never failed. Much more could be said of other vital organs, all under the control of the involuntary powers. In the face of all these facts, how strikingly true is the saying, "How fearfully and wonderfully we are made," for, in regard to the heart, we have but to note that its nerve supply springs principally from three little ganglia, and a few nerve fibres originating from the brain, spinal cord and sympathetic.

How delicate the source, how fragile and thread like the conductors; how readily implicated, how sudden and certain the result. Yet, in death from such a cause, how obscure the character of the lesion.

### INVOLUNTARY ACTS OF EVERY DAY LIFE.

For many of the following facts I am indebted to Dr. Pendleton, of Belfast, Maine, taken from a reprint of his annual address, before the State Medical Association, (June 28th, 1870.)

For a plausible explanation of the causes, sources of involuntary actions of man, I am indebted to a paper, by Hammond, entitled, "The Brain not the sole organ of the Mind," published in the Journal of Nervous and Mental Disease," Chicago, 1876.

Man sleeps, dreams, awakes and oft times masticates, works, swears, and prays involuntarily. Says Pendleton a day seldom passes when we do not find that we have not done some slight thing, without knowing it, such involuntary acts as that indicated by the boy's reply, "I didn't whistle, it whistled itself," and so from every stage of life, to the grandmother knitting, asleep in her chair, we have similar illustrations,—the girl at the sewing machine watches her work, but the treadle runs on from habit, not thought, yet her foot stops instantly when she changes her work.

> The woman singeth at her spinning wheel;
> A pleasant chant, ballad or bacorolle—
> She thinketh of her song, upon the whole,
> Far more than of her flax; and yet the reel
> Is full, and artfully, her fingers feel
> With quick adjustment, provident control,
> The fibres too subtly twisted to unroll,
> Out to a perfect thread—."

In the absent-minded person however, habit works independent of control, and takes advantage of his abstractions, to lead his feet into the ditch, while his head is among the stars; or lets him swallow the dice, and throw his glass of wine into the back-gammon board.

Huxley tells the story of a French Soldier, who was shot in the left parietal bone, and in consequence became paralyzed on the opposite side of the body. Recovering in two years, from the paralysis, he entered upon a double existence. In his normal life he was perfectly well, and a capital hospital attendant. For a day or two in each month, he passed into another life, without intimation. In this abnormal existence he was active, moved about as usual, and was to all appearances, the same person as in his normal state. He went through all the ordinary habits of his life, yet during what may be termed this artificial, unreal existence, he neither saw, tasted, heard or smelt; nor was he conscious of anything whatever, and there was only one sense in a state of activity, that of touch, which was exceedingly delicate. If an obstacle obstructed his way, he knocked against it, and went to one side. If he was pushed in any direction he went straight on.

Another illustration is that of an old mule that had spent its life grinding bark, and, when too old for service, was turned out to grass. At stated intervals, every day he was observed to leave grazing, and travel round and round an apple tree, in the pasture. We can find individuals or even nations, whose life is hardly less mechanical than that of the poor soldier, or the broken down mule.

### INVOLUNTARY THOUGHT.

But we need not limit ourselves to mere physical action. Recent experiments and investigations are rapidly bringing us to the conclusion, that a large number of the acts of the mind, as well as of the body, may be produced under a two-fold form; that there are two co-ordinate modes of mental activity, the one conscious and the other unconscious. We can no more stop thinking, than cease breathing. Since the time of Leibnitz the leading Metaphysician of Germany, and such as Hamilton and Mill, of England, have affirmed that the mind may undergo modifications of great importance, without being itself conscious of the process.

It is familiar to every one; that when we have been trying to recover a lost idea, and have abandoned the effort, some time after, when busily engaged upon some other subject, it comes to us unbidden. The best solution of a perplexing question is often obtained by an unconscious process, where the attention has been completely withdrawn. Speaking upon this subject, Dr. Holmes relates an anecdote of Lord Polkomente, a Scotch judge, who, being asked how he prepared himself for a judicial decision replied; "Ye see I first read a' the pleadings; and then, after letting them wamble wi' the toddy in my wame, two or three days, I gie my ain interlocutor."

When an author of a late work on "Democracy and Monarchy in France," was charged with a certain passage verbatim from De Tocqueville, he could only say; "I believe there is such a thing as unconscious memory." Every one has experienced the advantage of a night's sleep, in modifying the decisions of a previous day.

A dream has frequently supplied the clue to a labyrinth in which the mind was hopelessly lost during its hours of wakefulness. Carpenter mentions the case of an eminent judge, who, sleeping in a strange house, dreamed that lizards were crawling over him. He could not imagine what had originated such a dream, until in going into the apartment through which he had passed the previous evening, he noticed a mantel clock, on the base of which were figures of crawling lizards. This he must have unconsciously seen, and the sight must have left a trace on his brain, though it left no record in his memory. Dreaming often takes for its materials, traces left by occurrences long since past.

Galen was accustomed to attach much importance to the medical intelligence of dreams. He declares that a man dreamed that his left thigh was transformed into a marble stone, and within a short while thereafter, he actually lost the use of his member by a dead palsy. A wrestler dreamed that he was in a vessel filled with blood and so deep that the crown of his head was scarcely visible. Galen inferred that the man was in want of a liberal b'ood letting, and by that means the pleurisy from which he was suffering was cured. Genius has often confessed that its sleeping self, surpassed its waking self.

Now the best explanation for all these strange phenomena of involuntary acts, deeds and thoughts is to be found in the admirable reasoning of Hammond in his theory that "Brain is not the sole organ of the mind," when he argues that we have no evidence to show that the mind can exist independently of the nervous system. On the contrary, every fact in our possession bearing upon the question of their relation, goes to prove that where there is no nervous system there is no mind; and where there is injury or derangement of the nervous system, there is corresponding injury or derangement of the mind. When we inquire into the matter of the absolute and relative quantity of gray nerve tissue, we find that in this respect, man stands preeminent, and it is to this fact that he owes the great mental development which places him so far above all other living beings, for it is the gray tissue which originates mind.

The white nerve tissue, as is well known, serves only for the transmission of impressions and impulse, unless regard is paid to this point, we would certainly fall into a serious error, in determining the relation existing between the mind and the nervous system, but, having it in view, the connection is at once clear and well defined, there being no exception to the law, that the mental development is in direct proportion to the amount of gray matter entering into the composition of the nervous system, of any animal of whatever kind. But all the gray tissue of the nervous system is not confined to the brain. A large proportion of it is found in the ganglia of the sympathetic, and some other nerves, and an amount second, only to that of the brain in quantity; and indeed, in some animals a large amount is present as an integral constituent of the spinal cord. By the term mind I understand a force developed by nervous action. It bears the same relation to gray nerve tissue, that heat, electricity or light, do to chemical or mechanical action. Why mind should result from the functionation of gray nerve tissue is no more a mystery than the fact that the mixing of water with sulpheric acid develops heat, or the rubbing of a piece of sealing wax with a piece of silk causes the evolution of electricity. All are equally beyond our understanding. All are equally ultimate facts in science and speculations, in regard to the rationale of these all are equally vain and unprofitable.

All the manifestations of which the mind is capable, in its fullest development are embraced in four acts; perception, the intellect, the emotions, and the will. Either one of these may be exercised independently of the others. Thus an individual may have a perception without any intellectual, emotional, or volitional manifestation; and so the intellect, the emotions, or the will, may be brought into action without the necessary participation of each other. It is, however, clearly established that all mental processes, of any kind, have their origin in perception, and that an individual born without the ability to perceive, either from defects in the external organs of the senses, or of the central ganglia, by which impression on these organs are converted into perceptions, would be devoid of intellect, emotion and will; would be in fact, lower in mental development than the most degraded types of animated beings. but let us suppose a case of a man with a disease of the upper part of the spinal cord, of such a character as to prevent its conveying volitional impulses from the brain to the muscle of the lower limbs. Now, let the soles of the feet be tickled, and we will find that they are drawn away, and generally with much more force than when the brain is allowed to act. Such a movement is probably one of a true reflex character. It is spasmodic and indeterminate, being more extensive than is necessary. But let us go still further in our suppositions, and imagine that in such a case the mere drawing away of the foot was not sufficient to escape the irritation, and that the individual deliberatety lifted up the other foot in the attempt to remove the offending object, and that this action, not proving adequate, he made two or three leaps, in order to escape. What will we call these movements? Would they not be evidence of perception and will? Would they not be movements performed with a definite purpose, the very best possible under the circumstances, to escape from the irritation, even though the brain were unconscious of them. It must be remembered that consciousness is not the necessary accompaniment of volition.

Warm-blooded animals are, for many reasons, not suitable subjects for experiments, such as are required in the phenomena under consideration, but in some of the lower animals, as the frog for instance, we find those conditions present which fit them for such investigation. Thus, if the entire brain be removed from a frog, the animal will continue to perform those functions which are immediately connected with the maintainance of life. The heart beats, the stomach digests, and the glands of the body continue to elaborate the several secretions proper to them. These actions are immediately due to the sympathetic system, though they soon cease if the spinal cord be materially injured. These movements are calculated to excite astonishment to those who see them for the first time, and who have embraced the idea that all intelligence resides in the brain. For instance, if in such a frog, the web between the toes is pinched, the limb is immediately withdrawn; if the shoulder is scratched with a needle, the hind foot of the same side is passed to remove the instrument; if the animal is held up by one leg it struggles, if placed on its back, a position to which frogs have a great antipathy, it immediately turns over on its belly. If one foot be held firmly with a pair of forceps, the frog endeavors to draw it away. If unsuccessful it places the other foot against the instrument and pushes firmly in the effort to remove it. Still not succeeding it writhes the body from side to side, and makes a forward movement.

All these and even more complicated motions are performed by the decapitated alligator, and in fact may be witnessed to some extent in all animals. I have repeatedly seen, says Hammond, the headless body of the rattlesnake coil itself into a threatening attitude, and when irritated, strike its bleeding trunk against the offending body. Upon one occasion a teamster on the Western plains had decapitated one of these reptiles with his whip and while bending down to examine it more carefully was struck by it full in the face. So powerful was the shock to his nervous system, that he fainted and remained insensible for several minutes. According to Maine de Biran Perrault, reports that a viper, whose head had been cut off moved determinately towards its hole. This subject has been well studied by Dr. Towler, of New Orleans, Fluger, Paton, Onimus, and several others.

Now when we come to man and observe the experiments which are constantly being made for us, both in health and in disease, we cannot avoid placing the spinal cord much higher as a nerve centre than it is usually located by Physiologists.

In anni-cephalic monsters, we have interesting examples of the facts that the spinal cord is possessed of perception and volitional power. Syme describes one of these beings which had lived six months. Though very feeble, it had the faculty of sucking, and the several functions of the body appeared to be well performed. Its eyes clearly perceived the light, and it cried if the candle was extinguished. After death the cranium was opened, and there was found to be an entire absence of the cerebrum, the place of which was occupied by a quantity of sirous matter, contained in the arachnoid. The cerebellum and pons varolia were present.

Panizzat, of Pavia, reports the case of a male infant, which lived eighteen hours. Respiration was established but the child did not cry, nevertheless it was not insensible. Light impressed the eyes, as the pupils acted. A bitter juice put into the mouth was immediately rejected. Loud noises caused movement of the body. On post-mortem examination there was found no vestige of either cerebrum or cerebellum, but the medulla oblongata and pons varolia existed. There were no olfactory nerves; the optic nerves were atrophied, and the third and fourth pairs were wanting. All other cranial nerves were present.

In conclusion, Hammond deduces these propositions:

*First.* That of the mental faculties, perception and volition are seated in the spinal cord, as well as in the cerbral ganglia.

*Second.* That the cord is not probably capable of originating mental influence independently of sensorial impressions. The same may be said of the brain.

*Third.* That as memory is not an attribute of the mental influence evolved by the spinal cord, it requires, unlike the brain, a new impression, in order that mental force may be produced.

Dreams should be considered as involuntary acts of the brain, greatly influenced by the sympathetic, as the condition of the digestion modifies their character. A patient once applied to Dr. Abernethy in consequence of continually dreaming of his grandfather. "What do you eat so late for? What do you eat at bed-time?" asked the doctor; "Not much sir, only half a mince-pie." "Go," says Abernethy, "eat a whole one, and you will dream of your great grandfather."

If the sympathetic has so much to do with our dreams, we can then better account for the beautiful images produced by opium and other narcotics, stimulants, etc.

*First.* How belladonna dilates the pupil, how opium contracts it by their direct influence on the sympathetic, and by the sympathetic reflex action on the brain.

In my next paper, we will consider the diseases of the vasa-motor and some other important derangements of involuntary action.

